# SUV39H1-DNMT3A-mediated epigenetic regulation of Tim-3 and galectin-9 in the cervical cancer

**DOI:** 10.1186/s12935-020-01380-y

**Published:** 2020-07-20

**Authors:** Li Zhang, Sijuan Tian, Minyi Zhao, Ting Yang, Shimin Quan, Qing Yang, Lihua Song, Xiaofeng Yang

**Affiliations:** 1 Department of Gynecology and Obstetrics, The First Affiliated Hospital of Xi’an Jiaotong University, No.277 West Yanta Road, Xi’an, 710061 China; 2 School of Agriculture and Biology, Shanghai Jiao Tong University, Shanghai, 200240 China

**Keywords:** Cervical cancer, SUV39H1, H3K9me3, DNMT3A, Tim-3, Galectin-9

## Abstract

**Background:**

Methylation of histone 3 at lysine 9 (H3K9) and DNA methylation are epigenetic marks correlated with genes silencing. The tumor microenvironment significantly influences therapeutic responses and clinical outcomes. The epigenetic-regulation mechanism of the costimulatory factors Tim-3 and galectin-9 in cervical cancer remains unknown.

**Methods:**

The methylation status of *HAVCR2* and *LGALS9* were detected by MS-PCR in cervical cancer tissues and cell lines. The underlying molecular mechanism of SUV39H1-DNMT3A-Tim-3/galectin-9 regulation was elucidated using cervical cancer cell lines containing siRNA or/and over-expression system. Confirmation of the regulation of DNMT3A by SUV39H1 used ChIP-qPCR.

**Results:**

SUV39H1 up-regulates H3K9me3 expression at the *DNMT3A* promoter region, which in turn induced expression of DNMT3A in cervical cancer. In addition, the mechanistic studies indicate that DNMT3A mediates the epigenetic modulation of the *HAVCR2* and *LGALS9* genes by directly binding to their promoter regions in vitro. Moreover, in an in vivo assay, the expression profile of SUV39H1 up-regulates the level of H3K9me3 at the *DNMT3A* promoter region was found to correlate with Tim-3 and galectin-9 cellular expression level.

**Conclusion:**

These results indicate that SUV39H1-DNMT3A is a crucial Tim-3 and galectin-9 regulatory axis in cervical cancer.

## Background

Cervical cancer is the fourth common female malignancy worldwide [1]. In 2018, there was an estimated 569,847 new cases of cervical cancer and 311,365 deaths occurred worldwide [2]. High-risk human papillomavirus (HR-HPV) caused almost all cervical cancer [3]. Persistent HR-HPV infection caused chronic microenvironment changes of cervix. During carcinogenesis, over-adapted cancer cells eluded cytotoxic tumor infiltrating lymphocytes in a scenario of immune-tolerance driven by T-regulatory cells so that immune response is blocked [4]. T-cell immunoglobulin and mucin-domain containing-3 (Tim-3) negatively regulates Th1 immunity, once Tim-3 binds to its ligand galectin-9 could inhibit Th1 and Th17 responses by hampering their expansion, its mediating immune exhaustion in tumor microenvironment [5–7]. Tim-3^+^CD4^+^T cells represent the functional regulatory T cells which contribute to the formation of the immune-suppressive in human cervical cancer [8].

The epigenetic regulation of genes is critical for gene’s transcription [9]. DNA methylation is participated in gene expression changes that occur in cervical cancer [10]. Our previous study revealed that EZH2-H3K27me3-DNMT3A mediates the epigenetic regulation of the negative stimulatory molecules, Tim-3 and galectin-9 in cervical cancer which is associated with HPV18 infection [11]. Trimethylation of histone 3 lysine 9 (H3K9me3) at gene promoter regions is an important epigenetic mechanism that silences genes expression [12, 13] and SUV39H1 is H3K9me3-specific histone methyltransferase [14].

In present study, we identified an important role of histone and DNA methylation marks in regulating costimulatory factors Tim-3 and galectin-9 expression in cervical cancer. The relevant mechanism is mediated by the inhibition of Tim-3 and galectin-9 through recruiting DNMT3A to their promoter regions. SUV39H1 targeted DNMT3A by increasing the level of H3K9me3 at the *DNMT3A* promoter region so that repressed Tim-3 and galectin-9 expression by DNA methylation in cervical cancer. These results represent a significant step forward in understanding the contribution of SUV39H1 and DNMT3A to cervical cancer progression and providing a potential target for epigenetic-based cervical cancer therapy.

## Materials and methods

### Patients and samples

24 cervical cancer tissues, accordingly matched peri-carcinomatous tissues and 16 normal cervical tissues were obtained from the First Affiliated Hospital of Xi’an Jiaotong University between January 2014 and December 2017. All patients were diagnosed by two senior pathologists and none had received chemotherapy or radiotherapy prior to surgery. The cervical cancer samples were collected as previous described [15]. After the tissues were dissected, each sample was washed with sterilized PBS thrice and stored at − 80 °C. All procedures were performed on ice.

### Data mining

Oncomine database (www.oncomine.org) was used to detect the *HAVCR2* and *LGALS9* mRNA expression levels in cervical cancer and normal cervix tissues. The correlation between *HAVCR2* and *LGALS9* expression was studied by the data obtained from the GEPIA database (http://gepia.cancer-pku.cn/).

### Cell lines and culture conditions

The cervical cancer cell lines SiHa, HeLa and C33A were obtained from Cell Bank, Shanghai Institutes for Biological Sciences, Chinese Academy of Sciences, Shanghai. All cell lines were cultured in high glucose Dulbecco’s Modified Eagle’s Medium (DMEM) (HyClone, USA) supplemented with 10% fetal bovine serum (FBS) (Biological Industries, Israel) at 37 °C in an atmosphere of 5% CO_2_.

### Lentivirus vectors and stable expression cell lines construction

Lentiviral vector preparation of Plenti-CMV-puro-Dest vector containing SUV39H1 fragment. The SUV39H1 fragment was cloned from the genomic DNA of SiHa. DNA fragment treated with Kpn1 (TaKaRa, China) and Xho1 (TaKaRa, China) and then the target gene was linked to entry vector pENTR-MCS. Two-plasmid of Plenti-CMV-puro-Dest and pENTR-MCS recombination reactions were performed using LR Clonase II (Invitrogen, USA). Using Lip2000 (Invitrogen, USA) to transfect plasmid into SiHa and HeLa cell lines. The transduced cells were then selected by puromycin. Stably transduced cells were maintained in culture in the presence of puromycin. The cell lines were named SiHa-SUV39H1, HeLa-SUV39H1, successively. The expression level of SUV39H1 was determined by western blotting.

### RNA interference

SiHa and HeLa cell lines were transfected with scramble, SUV39H1 and DNMT3A specific siRNA (GenePharma, China), the following siRNA oligos for SUV39H1 and DNMT3A are listed in Table 1. The siRNAs respectively using X-tremeGENE siRNA Transfection Reagent (4476115001, Roche, Germany) and analyzed for SUV39H1 and DNMT3A expression levels by western blotting. All cell lines were named SUV39H1-siRNA and DNMT3A-siRNA, successively.

**Table 1:** Primer sequences

Name	Application	Sequence
*DNMT3A*-ChIP-F1	ChIP-qPCR	ATCATCAGTAGGGCGGGGTGGCCAC
*DNMT3A*-ChIP-R1	ChIP-qPCR	CTCCAATGCTTCCAGGTCCCTCCGT
*DNMT3A*-ChIP-F2	ChIP-qPCR	TTGGAGAACCTCCCGAAGGAAAACC
*DNMT3A*-ChIP-R2	ChIP-qPCR	GCCACCCTTTTAGCGTCACAGAACC
*DNMT3A*-ChIP-F3	ChIP-qPCR	CGTTGGGGGGGCGGGTGCTGGGCTG
*DNMT3A*-ChIP-R3	ChIP-qPCR	TGACTGGCACAGGACATGGCGTGCT
*DNMT3A*-ChIP-F4	ChIP-qPCR	CATGGGGAAGGAGAACAGCCCCCAC
*DNMT3A*-ChIP-R4	ChIP-qPCR	GCACTGGAAGACTGAAAGATTTCAT
*HAVCR2*-ChIP-F1	ChIP-qPCR	GTGGAAAAAATCTGTCACTTAGGGG
*HAVCR2*-ChIP-R1	ChIP-qPCR	ATTTTTAGTAGAGACGGGGTTTCTC
*HAVCR2*-ChIP-F2	ChIP-qPCR	CCTGTAATCCCAGCTACTCAGGAGG
*HAVCR2*-ChIP-R2	ChIP-qPCR	CTTGTTCAATGTGTGTACTTCCCAT
*HAVCR2*-ChIP-F3	ChIP-qPCR	CCCAATGCATTTAATGGCATAAATG
*HAVCR2*-ChIP-R3	ChIP-qPCR	CAGCCACACTCCCATAACTGAGGTA
*HAVCR2*-ChIP-F4	ChIP-qPCR	GGAACTCAACACTTTCTGATCATTC
*HAVCR2*-ChIP-R4	ChIP-qPCR	GACTTTGACCTTCAAACTTCCAACT
*LGALS9*-ChIP-F1	ChIP-qPCR	GGTAGAGTAAAATGTACAGATCCTG
*LGALS9*-ChIP-R1	ChIP-qPCR	GCGAGACCTTGTCTCTACTAAAAAT
*LGALS9*-ChIP-F2	ChIP-qPCR	TCAGCCTCCCAATGTGCTGAATTAC
*LGALS9*-ChIP-R2	ChIP-qPCR	CCAGATCCAAACTTGACTTGAAGTG
*LGALS9*-ChIP-F3	ChIP-qPCR	TCCTGTGGCCTAGCTCCTTTTTATT
*LGALS9*-ChIP-R3	ChIP-qPCR	AGAAAAACTGCTTGGTGAGTTGTAA
*LGALS9*-ChIP-F4	ChIP-qPCR	CACATATGTTTTCCTTTCTCTTGGG
*LGALS9*-ChIP-R4	ChIP-qPCR	ACACCTGTGGTCTCAGCTACATGGG
*HAVCR2*-ML	MS-PCR	TATAAAATGAGAAATTGGTCGGGCG
*HAVCR2*-MR	MS-PCR	TTACAAACATATACCACCACCCCGA
*HAVCR2*-UL	MS-PCR	GAAATTGGTTGGGTGTGGTGGTTAT
*HAVCR2*-UR	MS-PCR	TATACCACCACCCCAAATAATTTTA
*LGALS9*-*9*-ML	MS-PCR	TTTTCGAGATAGGTTTGCGATTTTG
*LGALS9*-*9*-MR	MS-PCR	AATACCGACACCCTTCAATCACCAC
*LGALS9*-*9*-UL	MS-PCR	GAGTTTTTGAGATAGGTTTGTGATT
*LGALS9*-*9*-UR	MS-PCR	ATACCAACACCCTTCAATCACCACA
*SUV39H1*-sense	Gene silencing	CCUUCGUGUACAUCAAUGATT
*SUV39H1*-anti-sense	Gene silencing	UCAUUGAUGUACACGAAGGTT
*DNMT3A*-sense	Gene silencing	GCCAAGGUCAUUGCAGGAATT
*DNMT3A*-anti-sense	Gene silencing	UUCCUGCAAUGACCUUGGCTT
Negative control-sense	Gene silencing	UUCUCCGAACGUGUCACGUTT
Negative control-sense	Gene silencing	ACGUGACACGUUCGGAGAATT

*ChIP* chromatin immunoprecipitation, *RT-PCR* reverse transcription-polymerase chain reaction, *F* forward primer, *R* backward primer, *MS-PCR* methylation-specific-polymerase chain reaction, *ML/UL* methylation/unmethylation forward primer, *MR/UR* methylation/unmethylation backward primer

### 5-Aza-2′-deoxycytidine treatment

1.0 × 10^5^/well SiHa, HeLa and C33A cells were cultured in 6-well plates in DMEM with 10% FBS, after 24 h, the medium was replaced with fresh medium containing 0 μM, 2.5 μM or 5 μM 5-Aza-2′-deoxycytidine (5-Aza-CdR) (Sigma, USA). The medium containing 5-Aza-CdR was replaced every 24 h during a 72-h period [15].

### DNA extraction, bisulfite modification and methylation-specific PCR (MS-PCR)

Genomic DNA was isolated from cells and tissues using TaKaRa Mini BEST Universal Genomic DNA Extraction Kit (TaKaRa, China) according to the manufacturer’s instruction. DNA modification was done as previous described [15], in briefly, 500 ng genomic DNA was bisulfite-modified by EZ DNA Methylation-Gold™ Kit (Zymo Research, USA). Modified DNA templates were used for MS‑PCR with Zymo TaqTM PreMix (E2003, Zymo Research, USA) following the instructions of the manufacturer. The primers used in MS-PCR are listed in Table 1. The annealing temperature for the methylated primers of *HAVCR2* and *LGALS9* were 60 °C and 60 °C while that for the unmethylated primers were 55 °C and 56.3 °C. The MS-PCR products were separated on a 2% agarose gel, stained with Gelview and visualized under ultraviolet illumination (Bio-Rad, USA). Methylation level was calculated by the ratio of methylated and unmethylated levels. Grey value of each band represented its relative expression and was measured by Image J Software. Each reaction was performed in triplicate.

### Western blotting analysis

Cells were treated with RIPA Lysis Buffer which contained 1 mM PIC and 1 mM PMSF. Proteins were separated by SDS-PAGE and electroblotted onto PVDF membrane (Millipore, USA), then blocked 1 h at room temperature in 5% skim milk and incubated with primary antibodies for overnight at 4 °C followed by HRP conjugated secondary antibodies for 1 h at room temperature. The secondary antibodies are as follows: HRP-conjugated rabbit anti-mouse IgG (1:5000 dilution, D110273-0100, BBI Life Sciences, China), HRP-conjugated goat anti-rabbit IgG (1:5000 dilution, 31460, PIONEER, China). Chemiluminescence signal was detected following incubation with enhanced chemiluminescence reagent (Millipore, USA). Grey value of each band was measured with Image J Software. The primary antibodies are listed in Table 2.

**Table 2:** Primary antibodies

Antibody	Source	Dilution	Cat number	Application
H3K9me3	Cell Signaling Technology	1:50/1:1000	13969	ChIP, WB
SUV39H1	Cell Signaling Technology	1:1000	8729	WB
Tim-3	Abcam	1:50	ab47997	IF
Tim-3	Abcam	1:250/1:250	ab185703	WB, IHC
Galectin-9	Abcam	1:200	ab123712	WB
Galectin-9	Abcam	1:250/1:200	ab69630	IF, IHC
DNMT3A	Abcam	1:50/1:250	ab13537	ChIP, WB
IgG	Cell Signaling Technology	1:500	2729	ChIP
Histone H3	Cell Signaling Technology	1:50	4620	ChIP
β-actin	TransGen Biotech	1:500	HC201-01	WB

*ChIP* chromatin immunoprecipitation, *WB* western blotting, *IF* immunofluorescence, *IHC* immunohistochemical

### Chromatin immunoprecipitation (ChIP) assay and ChIP-qPCR

ChIP assays were carried out using the Simple ChIP Enzymatic Chromatin IP Kit (Cell Signaling Technology, USA) according to the manufacturer’s instructions. The *DNMT3A*, *HAVCR2* and *LGALS9* promoter regions were detected by qPCR using promoter DNA-specific primers. The primers are listed in Table 1. We used the cycle threshold (CT) as the representative point. The relative expression of genes in each group (fold-change compared with control) was calculated using the formula: RQ = 2^−∆∆Ct^. Each reaction was performed in triplicate. The antibodies are listed in Table 2.

### Immunofluorescence staining

SiHa and HeLa cells were incubated with primary antibodies overnight at 4 °C. After washing three times, the cells were incubated with R-conjugated Donkey anti-rabbit IgG (1:200 dilution, sc-2095, Santa Cruz Biotechnology, USA) and FITC-conjugated Donkey anti-goat IgG (1:200 dilution, EK033, Zhuangzhibio, China) for 1 h at room temperature. Finally, DAPI Fluoromount-G (SouthernBiotech, USA) was used to counterstain the cell nuclei. The fluorescent was detected, and images were taken by Leica inverted fluorescence microscope. The primary antibodies are listed in Table 2.

### Immunohistochemical staining

Human cervical cancer specimens were fixed with neutral formalin, embedded in paraffin, and sectioned at a thickness of 4 μm. Sections were deparaffinized in xylene and rehydrated in a graded alcohol series. Antigen retrieval was performed using 0.01-M citrate buffer and 2 min of boiling. Hydrogen peroxide was applied to block endogenous peroxidase activity, and then sections were incubated with normal goat serum (SP-9001, Zsbio, China) to block nonspecific protein binding. Sections stained with primary antibody for Tim-3 and galectin-9 were incubated overnight at 4 °C. Sections were stained in parallel with PBS as a negative control. Tim-3 and galectin-9 expression were then detected using DAB, and slides were counterstained with hematoxylin. Slides were view at 400× magnification. The primary antibodies are listed in Table 2.

### Xenograft mouse model

Female BALB/c nude mice (4-week-old) used in this study and were maintained in a specific-pathogen-free (SPF) condition facility. Mouse injected subcutaneously with 1 × 10^7^ SiHa-SUV39H1/HeLa-SUV39H1 cells were randomly divided into four groups when tumor volumes were around 100 mm^3^: (1/2) SiHa-mock/HeLa-mock control groups; (3/4) SiHa-SUV39H1/HeLa-SUV39H1 groups. Two diameters of the individual tumor were measured by electronic slide caliper every 2 days. Tumor volume was calculated using the following formula: tumor volume (mm^3^) = 0.5 × length × width^2^. Mice were monitored for 21 days, at which time mice were euthanized and tumors and organs were extracted.

### Statistical analysis

Statistical analyses were performed using GraphPad Prism 7 software (GraphPad Software, USA). Paired t test and one-way ANOVA analysis were carried out on samples within groups. The *P* value of < 0.05 was considered statistically significant. The *P* values are represented as **P < 0.01, *P < 0.05. The data are presented as mean ± standard error of the mean (SEM). All experiments were independently repeated at least thrice, with consistent results.

## Results

### Tim-3 and galectin-9 expression were increased due to genes methylation level decreased in cervical cancer

**Fig. 1:**
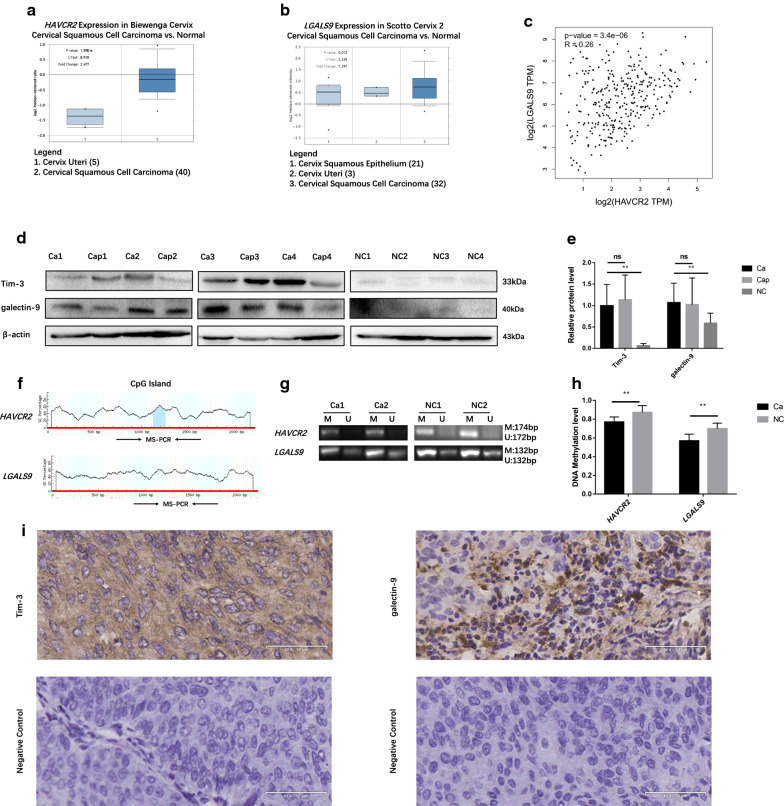
The expression of Tim-3 and galectin-9 in cervical cancer tissues and the genes methylation level. **a**, **b** An overview of mRNA levels of *HAVCR2* and *LGALS9* in cervical cancer based on Oncomine database. **c** The correction between *HAVCR2* and *LGALS9* in cervical cancer, analyzed by GEPIA database. **d**, **e** The protein levels of Tim-3 and galectin-9 in cervical cancer (Ca) (n = 24), para-carcinoma (Cap) (n = 24) and normal cervical tissues (NC) (n = 16) detected by western blotting. Blot images of four representative samples are shown from each group. **f** Predicted CpG islands in the promoter regions of *HAVCR2* and *LGALS9*. Numbers indicate the positions in bp relative to the transcription start site. The blue region represents the CpG islands and the red vertical bars are the CpG loci in these input sequences. **g**, **h** Methylation level of *HAVCR2* and *LGALS9* promoter regions in cervical cancer (Ca) (n = 9) and NC (n = 9) detected by MS-PCR. (*M* methylated, *U* unmethylated); methylated and unmethylated levels of genes were quantified as M/M + U% and U/M + U%, respectively. **i** Positive immunohistochemistry staining of Tim-3 and galectin-9 and negative control in representative cervical cancer samples. Magnification, ×400. **P < 0.01, *ns* not significant

Using the Oncomine databases (https://www.oncomine.org/), the mRNA expression levels of *HAVCR2* and *LGALS9* were compared between cervical cancer and normal cervical samples. The results indicated that the expression levels of *HAVCR2* and *LGALS9* were all higher in cervical cancer than in normal cervical samples (Fig. 1a, b). Furthermore, *HAVCR2* was positively corrected with *LGALS9* (R = 0.26, *P* < 0.05) based on Gene Expression Profiling Interactive Analysis (GEPIA) dataset (http://gepia.cancer-pku.cn/) (Fig. 1c). Hence *HAVCR2* has a positive correlation with *LGALS9* in cervical cancer. The expression of Tim-3 and galectin-9 protein in cancer tissues were higher than in normal cervix tissues (Fig. 1d, e). The detail data of patients’ clinicopathological is shown in Table 3.

**Table 3:** Patients’ clinicopathological details (n = 24)

Item	No.
Age	
≤ 44	10
> 44	14
Clinical stages	
Ia	0
Ib	8
IIa	11
IIb	5
Pathological pattern	
Squamous cell carcinoma	21
Adenocarcinoma	3
Pathological grading	
I	1
II	19
III	4
Lymph nodes metastasis	
Yes	3
No	21
HPV infection	
Positive	20
Negative	4

The online software “MethPrimer” (http://www.urogene.org/methprimer/) profiled CpG island in the region that was located from − 2000 to − 200 bp upstream from ATG, the transcription starts site (TSS) in the *HAVCR2* and *LGALS9* promoter regions respectively (Fig. 1f). One pair of primer was designed to amplify the genes promoter regions respectively. *HAVCR2* and *LGALS9* promoter regions in cervical cancer tissues displayed hypermethylation status in normal cervical tissues (Fig. 1g, h), possibly leading to the inhibition of transcriptional activity of genes in normal cervical tissues. Immunohistochemistry results revealed that both Tim-3 and galectin-9 expressed in tumor cells of cervical cancer tissues, and galectin-9 also showed evident staining in peritumoral inflammatory infiltrate of cervical cancer tissues (Fig. 1i).

### Tim-3 and galectin-9 expression were reversed by alter the methylation status in the promoter regions of *HAVCR2* and *LGALS9* in cervical cancer cells

**Fig. 2:**
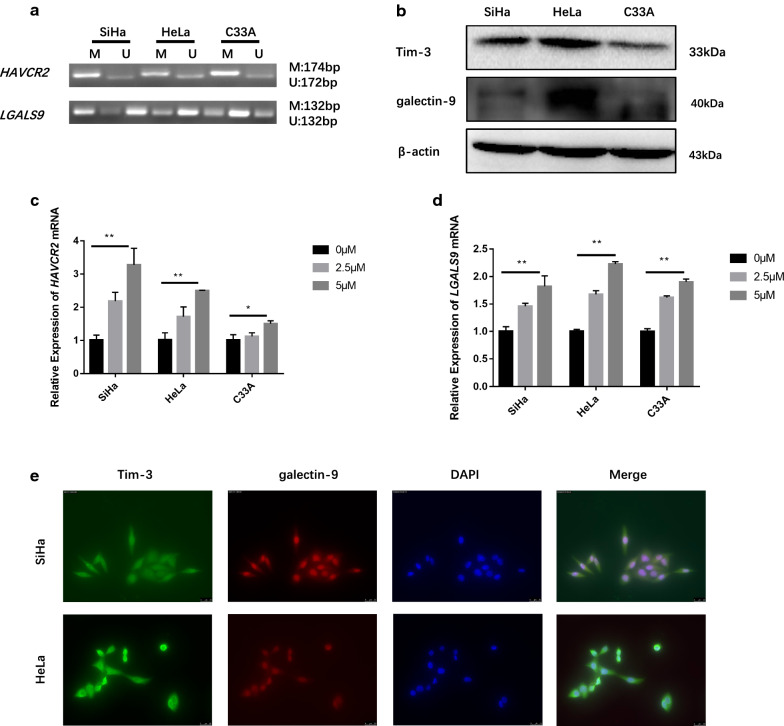
Methylation status in the promoter regions of *HAVCR2* and *LGALS9* in cervical cancer cells. **a** Detection of *HAVCR2* and *LGALS9* methylation status by MS-PCR in SiHa, Hela and C33A cells; (*M* methylated, *U* unmethylated). **b** Tim-3 and galectin-9 expressed in SiHa, HeLa and C33A cells detected by western blotting. **c**, **d** Relative expression of *HAVCR2* and *LGALS9* mRNA in SiHa, HeLa and C33A cells after treatment with different concentrations of 5-Aza-CdR. **e** Representative immunofluorescence staining of Tim-3 and galectin-9 expression in SiHa and HeLa cells. *P < 0.05, **P < 0.01

*HAVCR2* and *LGALS9* promoter regions in SiHa, HeLa and C33A cells were all partially methylated (Fig. 2a). The Tim-3 and galectin-9 were both expressed in SiHa, HeLa and C33A cells (Fig. 2b). To identify whether the methylation status in the promoter regions regulate the expression of these genes at the transcription level, the mRNA expression levels of *HAVCR2* and *LGALS9* in SiHa, HeLa and C33A cell lines after treatment with DNA demethylation reagent 5-Aza-CdR were detected. The results suggested that the expression levels of *HAVCR2* and *LGALS9* mRNA in SiHa, HeLa and C33A cells were increased in dose-dependent manner after cellular DNA demethylation (Fig. 2c, d). It illustrated the Tim-3 and galectin-9 expression were reversed by 5-Aza-CdR, which promoted the expression of these genes at the transcriptional level. Immunofluorescence assay showed that the SiHa and HeLa cells both staining cytoplasm and nuclear Tim-3 and galectin-9 (Fig. 2e).

### Tim-3 and galectin-9 expression were repressed by DNMT3A through mediated DNA methylation

**Fig. 3:**
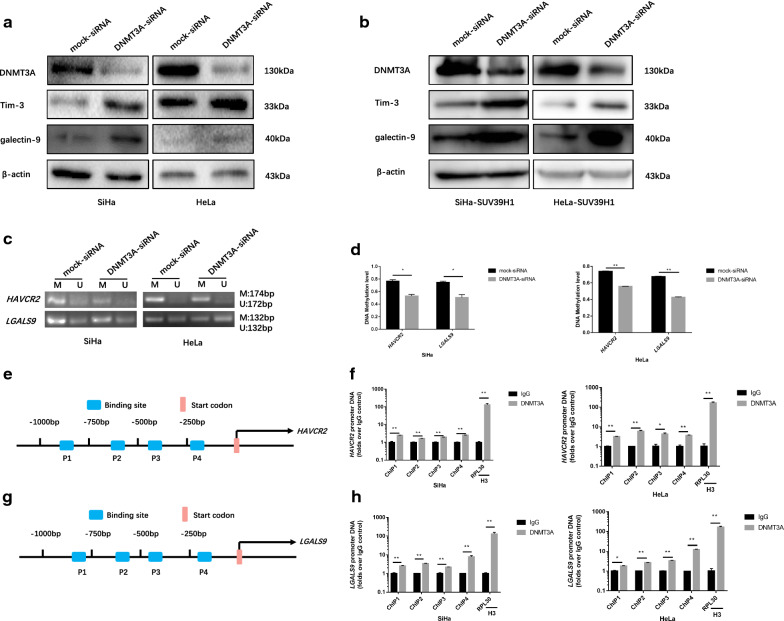
Tim-3 and galectin-9 were repressed by DNMT3A mediated DNA methylation. **a** Western blotting analysis of SiHa and HeLa cells knocked-down DNMT3A against Tim-3, galectin-9 and DNMT3A. **b** Detection of the expression of DNMT3A and Tim-3, galectin-9 in DNMT3A specific siRNA transfected overexpressed SUV39H1 SiHa and HeLa cells by western blotting. **c** The methylation level of *HAVCR2* and *LGALS9* promoter regions were monitored by MS-PCR in DNMT3A knockdown SiHa and HeLa cells. **d** Methylated and unmethylated levels were quantified as M/M + U% and U/M + U%, respectively. **e**, **g** Schematic representation of the four regions of the *HAVCR2* and *LGALS9* promoter regions amplified in the chromatin immunoprecipitation (ChIP)‑quantitative PCR (qPCR) experiment. **f**, **h** Chromatin was cross-linked, fragmented and immunoprecipitated with either IgG (mock) or anti-DNMT3A ChIP-grade antibody and the purified DNA was used to amplify with respective primer pairs for indicated four regions in the *HAVCR2* and *LGALS9* promoter regions in qPCR. The enrichment of DNMT3A on *HAVCR2* and *LGALS9* promoter regions relative to IgG in SiHa and HeLa cells, and H3 against *RPL30* was used as positive control. *P < 0.05, **P < 0.01

The study found that knocking-down DNMT3A activated Tim-3 and galectin-9 expression (Fig. 3a, b), accompanied by decreased DNA methylation level of the genes’ promoter regions in SiHa and HeLa cells (Fig. 3c, d), suggesting that DNMT3A play a critical role in Tim-3 and galectin-9 expression regulation. The ChIP analysis revealed enhanced binding of DNMT3A (Fig. 3f, h) to the *HAVCR2* and *LGALS9* promoter regions (Fig. 3e, g) in SiHa and HeLa cells. Altogether, these results suggested that DNMT3A-mediated DNA methylation contributes to the transcriptional silencing of *HAVCR2* and *LGALS9*.

### SUV39H1 mediated DNMT3A expression through up-regulating H3K9me3 in cervical cancer cells

**Fig. 4:**
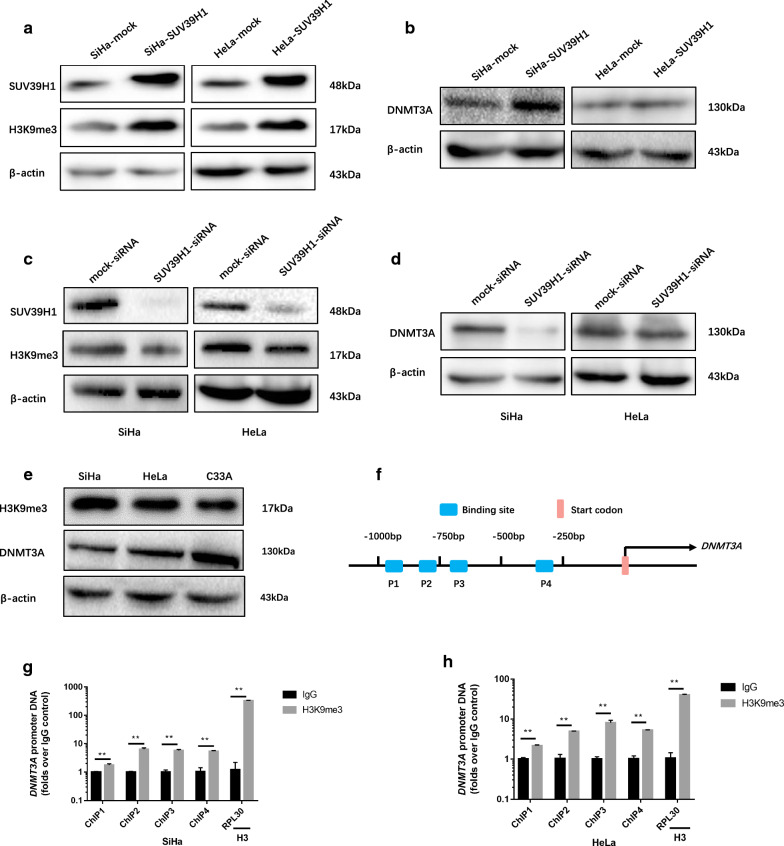
SUV39H1 increased DNMT3A expression in cervical cancer cells. **a**, **b** Detection the expression of SUV39H1, H3K9me3 and DNMT3A in SiHa and HeLa cells which overexpressed SUV39H1 by western blotting. **c**, **d** Western blotting analysis of SUV39H1, H3K9me3 and DNMT3A in SiHa and HeLa cells 48 h after transfection with siRNAs against SUV39H1. **e** Detection of the expression of H3K9me3 and DNMT3A in SiHa and HeLa cells by western blotting. **f** Schematic representation of the four regions of the *DNMT3A* promoter amplified in the ChIP-qPCR experiment. **g**, **h** Chromatin was cross-linked, fragmented and immunoprecipitated with either IgG (mock) or anti-H3K9me3 ChIP-grade antibody and the purified DNA was used to amplify with respective primer pairs for indicated four regions in the *DNMT3A* promoter in qPCR. The enrichment of H3K9me3 on *DNMT3A* promoter relative to IgG in SiHa and HeLa cells, and H3 against *RPL30* was used as positive control. **P < 0.01

SUV39H1 is the histone methyltransferase (HMTase) of histone H3 lysine 9 trimethylation (H3K9me3) [16]. SUV39H1 recognizes trimethylated H3K9 (H3K9me3) via its chromodomain (CD), and enriched H3K9me3 afterwards [17]. The H3K9me3 and DNMT3A both expressed in SiHa, HeLa and C33A cells (Fig. 4e).

As shown in Fig. 4b, overexpressed SUV39H1 in SiHa and HeLa cells (Fig. 4a) significantly increased H3K9me3 and DNMT3A expression levels. Knocking-down SUV39H1 expression in SiHa and HeLa cells displayed dramatically down-regulation expression of H3K9me3 and DNMT3A (Fig. 4c, d). Collectively, these results indicated that SUV39H1 participate in the regulation of DNMT3A through changing H3K9me3 expression level in cervical cancer cells.

In a screening for epigenetic mechanism that regulating DNMT3A expression, ChIP analysis revealed that the SiHa and HeLa cells exhibit the highest H3K9me3 level in *DNMT3A* promoter region which upstream of the transcription initiation region (Fig. 4f). H3K9me3 expression level up-regulated on the − 1000 to + 1 region of the promoter region of *DNMT3A* (Fig. 4g, h). Taken together, above results revealed that SUV39H1 regulated the expression of DNMT3A through elevating H3K9me3 level at the *DNMT3A* promoter in cervical cancer cells.

### The methylation status of *HAVCR2* and *LGALS9* affected by SUV39H1 in cervical cancer cells

**Fig. 5:**
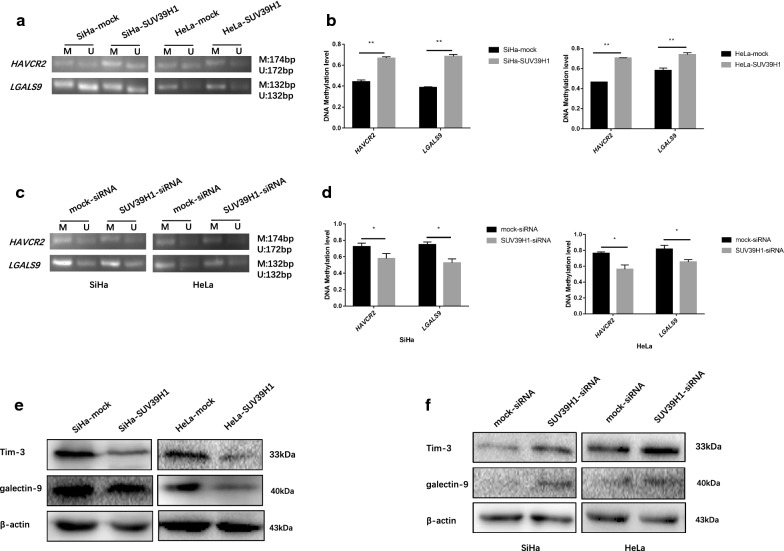
SUV39H1 changed *HAVCR2* and *LGALS9* methylation level in cervical cancer cells. **a**, **c** The methylation level of *HAVCR2* and *LGALS9* promoter regions were monitored by MS-PCR in SUV39H1 overexpressed and knocked-down SiHa and HeLa cells (*M* methylated, *U* unmethylated). **b**, **d** Methylated and unmethylated levels were quantified as M/M + U% and U/M + U%, respectively. **e**, **f** Western blotting analysis of SiHa and HeLa cells overexpressed and knocked-down SUV39H1 against Tim-3 and galectin-9. *P < 0.05, **P < 0.01

After have been determining the baseline levels of DNA methylation on *HAVCR2* and *LGALS9* promoter regions among cervical cancer cell line, evaluation whether SUV39H1 mediated DNA methylation through DNMT3A is required for *HAVCR2* and *LGALS9* transcription. The results showed that overexpressed SUV39H1 increased the methylation levels at the *HAVCR2* and *LGALS9* promoter regions (Fig. 5a, b), these changed methylation levels contributed to the decrease of Tim-3 and galectin-9 expression among overexpressed SUV39H1 in cell lines (Fig. 5e). SUV39H1-knocked-down cells showed the opposite results (Fig. 5c, d, f). To sum up, it was indicated that changing histone modification precede the alternation DNA methylation level of *HAVCR2* and *LGALS9*, indicating that SUV39H1 participated in changing the methylation status of *HAVCR2* and *LGALS9* by elevating the DNMT3A expression, so as to their transcriptional activity altered.

### SUV39H1 mediated Tim-3 and galectin-9 expression through DNA methylation in vivo

**Fig. 6:**
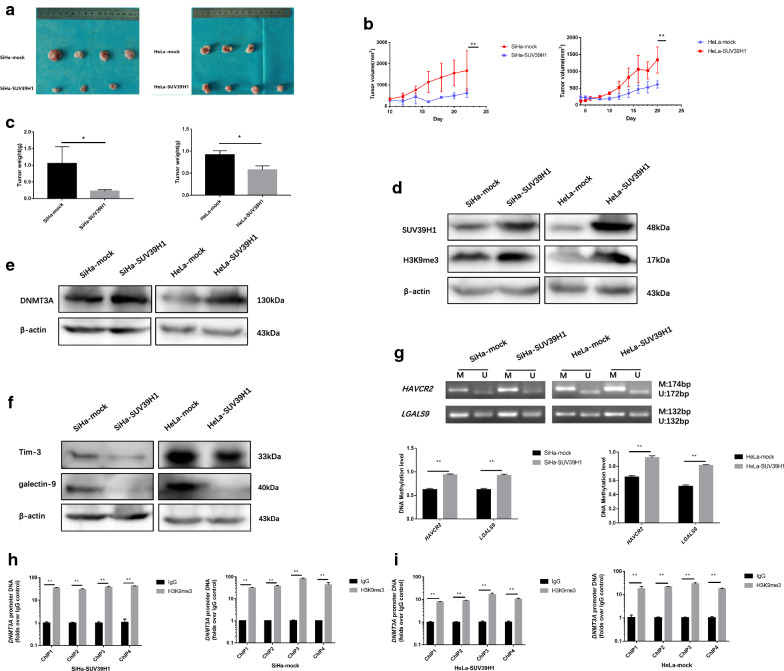
SUV39H1 mediated Tim-3 and galectin-9 expression through DNA methylation in vivo. **a** SiHa-SUV39H1 and HeLa-SUV39H1 tumor xenografts in nude mice. **b** Tumors formed from SiHa-SUV39H1 and HeLa-SUV39H1 cells as well as tumor growth curves. **c** The tumors weight formed from SiHa-SUV39H1 and HeLa-SUV39H1. **d**–**f** Western blotting results of SUV39H1, H3K9me3, DNMT3A, Tim-3 and galectin-9 in SiHa-SUV39H1 and HeLa-SUV39H1 cells formed tumors. **g** The methylation level of *HAVCR2* and *LGALS9* promoter regions were monitored by MS-PCR in tumor tissues. **h**, **i** Chromatin was cross‑linked, fragmented and immunoprecipitated with either IgG (mock) or anti‑H3K9me3 ChIP‑grade antibody and the purified DNA was used to amplify with respective primer pairs for the indicated four regions in the *DNMT3A* promoter in qPCR. The enrichment of H3K9me3 on *DNMT3A* promoter relative to IgG in tumor tissues. *P < 0.05, **P < 0.01

For the purpose of investigating SUV39H1 mediated the costimulatory factors Tim-3 and galectin-9 expression through DNA methylation in vivo, we generated SiHa-SUV39H1 and HeLa-SUV39H1 tumor xenografts in nude mice. As shown in Fig. 6a, tumors formed from the SiHa-mock and HeLa-mock cells (Fig. 6b, c) grew faster than those formed from the SiHa-SUV39H1 and HeLa-SUV39H1 cells, respectively. These data indicated that up-regulating SUV39H1 inhibited the tumor growth in SiHa and HeLa cells.

To determine SUV39H1 mediated Tim-3 and galectin-9 expression through DNA methylation in vivo, the levels of H3K9me3, DNMT3A, Tim-3 and galectin-9 in the tumor xenograft tissues were examined by western blotting. As shown in Fig. 6d–f, the expression of DNMT3A increased significantly when SUV39H1 overexpressed, followed by the down-regulation of Tim-3 and galectin-9 in SiHa-SUV39H1 and HeLa-SUV39H1 cells derived tumors. SUV39H1 overexpression significantly up-regulated the methylation level of *HAVCR2* and *LGALS9* in tumor tissues (Fig. 6g). ChIP analysis revealed that SUV39H1 regulated the expression of DNMT3A by improving H3K9me3 level acting on the − 1000 to + 1 region of the promoter region of *DNMT3A* (Fig. 6h, i) in tumor tissues. These results indicated that SUV39H1 precede the changes in DNA methylation. All the results in xenograft tissues were consistent with those in vitro, illustrated that similar SUV39H1 mediated Tim-3 and galectin-9 expression through DNA methylation in vivo.

### H3K9me3 expression was independent from HR-HPV oncogenes

**Fig. 7:**
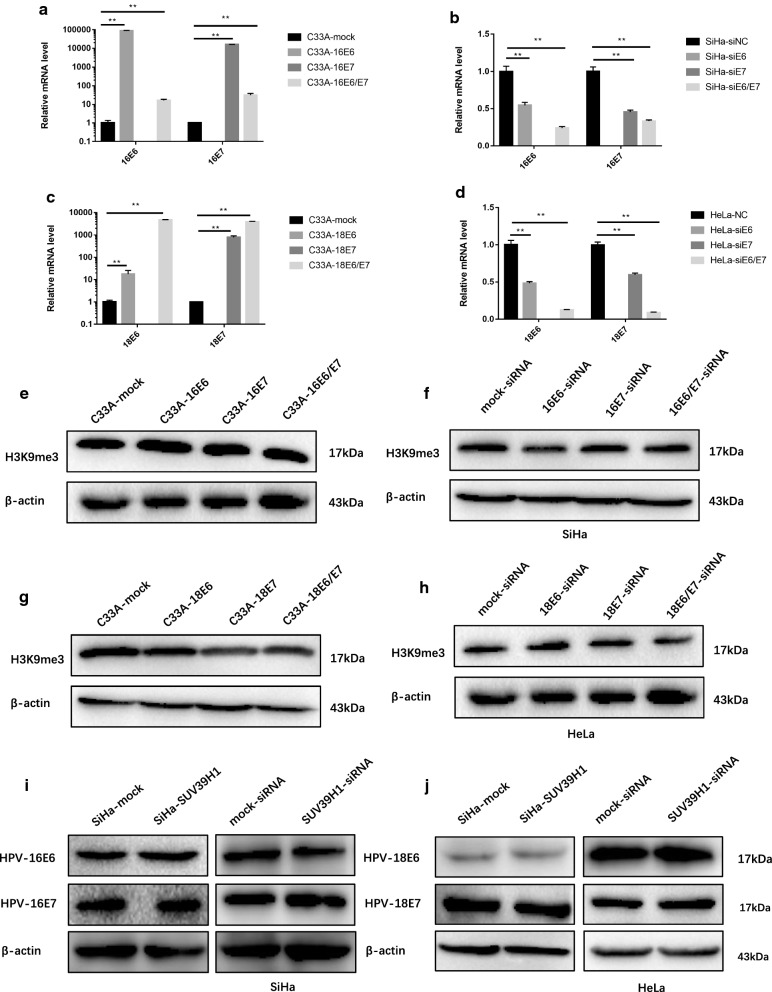
HR-HPV E6/E7 wasn’t participate in H3K9me3 mediated DNA methylation in cervical cancer. **a**–**d** mRNA levels in of HPV16 and 18 *E6*/*E7*-overexpressed C33A cells and of HPV16 and 18 *E6*/*E7*-knocked-down SiHa and HeLa cells. **e**, **f** Detection of the expression of H3K9me3 in HPV16 E6/E7 overexpressed C33A cells or knocked-down SiHa cells by western blotting. **g**, **h** Detection of the expression of H3K9me3 in HPV18 E6/E7 overexpressed C33A cells or knocked-down HeLa cells by western blotting. **i** HPV16 E6/E7 expression in overexpressed or knocked-down SUV39H1 in SiHa cells. **j** HPV18 E6/E7 expression in overexpressed or knocked-down SUV39H1 in HeLa cells

As persistent HR-HPV infection contributes to almost all cervical cancer cases [18], we attempted to explore whether HR-HPV oncogenes E6 and E7 participated in SUV39H1 mediated DNA methylation in SiHa and HeLa cells. there was no difference in the expression level of H3K9me3 in cervical cancer cells SiHa, HeLa and C33A (Fig. 4e). Expression of HPV16/18 E6 and E7 oncogenes were detected by western blotting in overexpressed or knocked-down SUV39H1 SiHa and HeLa cells respectively. As show in Fig. 7i, j, the expression level of HR-HPV oncogenes E6 and E7 were no difference between overexpressed or knocked-down SUV39H1 SiHa and HeLa cells with control. In the following studies we transiently overexpressed or knocked-down HPV16/18 E6 and E7 in cervical cancer cells for further illustration (Fig. 7a–d). The results showed that the level of H3K9me3 was not changed in overexpressed or knocked-down HPV16/18 E6 and E7 cells compared with control (Fig. 7e–h).

Taken together, our data suggested that H3K9me3 expression was independent from HR-HPV oncogene E6 and E7 in cervical cancer.

## Discussion

Aberrant DNA methylation plays an important role in cervical cancer carcinogenesis, which causes silencing of certain genes [19, 20]. Epigenetic modification also plays a crucial part in regulating immune cell differentiation [21, 22]. The methylation status of immune genes affects the tumor immune response in the tumor microenvironment (TME) [23, 24]. The study showed that reduced activity of DNMTs in CD4^+^Tregs was accompanied by demethylation of the forkhead box P3 (*FOXP3*) gene promoter and downregulation of immune responses in the TME [25]. Hypermethylation associated SMAD family member 3 (*SMAD3*) silencing in CAFs, which was associated with aberrant response to exogenous TGF-β1 [26]. Here, we identified a novel function of SUV39H1 regulates DNMT3A expression through elevating H3K9me3 level at the *DNMT3A* promoter region, which could mediate Tim-3 and galectin-9 expression through DNA methylation in cervical cancer. Tim-3 and galectin-9 are overexpressed in cervical cancer tissues, this biological effect is mediated through the aberrant epigenetic of Tim-3 and galectin-9, which is facilitated by the recruitment of DNMT3A to their promoter regions. Meanwhile, SUV39H1 contributes to Tim-3 and galectin-9 regulation by up-regulating the H3K9me3 level at the *DNMT3A* promoter region.

The study found that Tim-3 and galectin-9 were overexpressed in cervical cancer tissues related to promoter regions of *HAVCR2* and *LGALS9* were hypo-methylated, and they were partial methylated in cervical cancer cells, indicating DNA methylation mediating costimulatory factors Tim-3 and galectin-9 in cervical cancer cells. DNA methylation-based gene silencing in cancer [27]. DNMT3A involved in the induction of genes expression by directly binding to the *HAVCR2* and *LGALS9* promoter regions, suggested that DNMT3A participate in the epigenetic regulation of *HAVCR2* and *LGALS9* in cervical cancer. Knocked-down DNMT3A in cervical cancer cells caused a decreasing of *HAVCR2* and *LGALS9* methylation level, accompanied by the expression of Tim-3 and galectin-9 elevated.

H3K9me3 and DNA methylation leading to depression of a collection of genes [28, 29]. The changes in histone modification precede the alterations in DNA methylation [30, 31]. Up-regulation of SUV39H1 could promote DNMT3A expression, ChIP results demonstrated H3K9me3 expression level elevated at the *DNMT3A* promoter region to adjust its expression. These results suggested that SUV39H1 facilitates the expression of DNMT3A in cervical cancer. Overexpression SUV39H1 also associated with increased methylation level of *HAVCR2* and *LGALS9* which in turn caused Tim-3 and galectin-9 expression decreased in cervical cancer. SUV39H1 may be a prerequisite for promoter DNA methylation by recruiting DNMT3A, they cooperatively orchestrate epigenetic modification at the gene promoter regions of *HAVCR2* and *LGALS9*. But HPV16 or 18 oncogenes didn’t affect the expression of H3K9me3 in cervical cancer cells.

The epigenetic regulation caused elevated expression of costimulatory factors Tim-3 and galectin-9 in cancer cells, and the abnormal secreted Tim-3 and galectin-9 by tumor cells lead to tumor microenvironment immune imbalance, thereby promoting the development of cervical cancer. We provide evidence for SUV39H1 as a potential therapeutic target, which could decrease negative immune factors like Tim-3 and galectin-9. Activation the expression of SUV39H1 may potentially be an effective approach to increase the efficacy of immune cells against cervical cancer.

## Conclusion

**Fig. 8:**
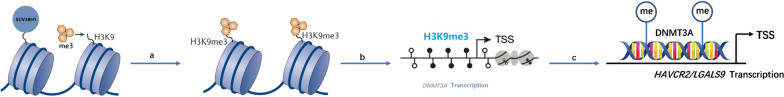
The pathway of SUV39H1 regulated Tim-3 and galectin-9 expression through DNA methylation. **a** SUV39H1 mediated H3K9 methylation. **b** The expression level of H3K9me3 increased at the *DNMT3A* promoter region so that its expression up-regulated. **c** DNMT3A directly bind to *HAVCR2* and *LGALS9* promoter regions respectively to increase their methylation level so that their transcriptional activity decreased

In summary, the present study highlights the role of SUV39H1 and DNMT3A in the DNA methylation regulation of Tim-3 and galectin-9 in cervical cancer (Fig. 8). We provide a potential direction in exploring the relationship between SUV39H1 and DNMT3A. These findings add diverse roles and mechanistic insight into our understanding of crosstalk of SUV39H1 with DNMT3A.

## Data Availability

The dataset analyzed during the current study are publicly available from the online database: GEPIA database (http://gepia.cancer‑pku.cn/) and Oncomine database (www.oncomine.org).
